# Breeding-assisted genomics: Applying meta-GWAS for milling and baking quality in CIMMYT wheat breeding program

**DOI:** 10.1371/journal.pone.0204757

**Published:** 2018-11-29

**Authors:** Sarah D. Battenfield, Jaime L. Sheridan, Luciano D. C. E. Silva, Kelci J. Miclaus, Susanne Dreisigacker, Russell D. Wolfinger, Roberto J. Peña, Ravi P. Singh, Eric W. Jackson, Allan. K. Fritz, Carlos Guzmán, Jesse A. Poland

**Affiliations:** 1 AgriPro Wheat, Syngenta, Junction City, Kansas, United States of America; 2 General Mills, Crop Bioscience Division, Manhattan, Kansas, United States of America; 3 SAS Institute Inc., JMP-Genomics Division, Cary, North Carolina, United States of America; 4 International Maize and Wheat Improvement Center, Mexico, D.F., Mexico; 5 25:2 Solutions, Rockford, Minnesota, United States of America; 6 Kansas State University, Department of Agronomy, Manhattan, Kansas, United States of America; 7 Kansas State University, Department of Plant Pathology, Manhattan, Kansas, United States of America; Institute of Genetics and Developmental Biology Chinese Academy of Sciences, CHINA

## Abstract

One of the biggest challenges for genetic studies on natural or unstructured populations is the unbalanced datasets where individuals are measured at different times and environments. This problem is also common in crop and animal breeding where many individuals are only evaluated for a single year and large but unbalanced datasets can be generated over multiple years. Many wheat breeding programs have focused on increasing bread wheat (*Triticum aestivum* L.) yield, but processing and end-use quality are critical components when considering its use in feeding the rising population of the next century. The challenges with end-use quality trait improvements are high cost and seed amounts for testing, the latter making selection in early breeding populations impossible. Here we describe a novel approach to identify marker-trait associations within a breeding program using a meta-genome wide association study (GWAS), which combines GWAS analysis from multi-year unbalanced breeding nurseries, in a manner reflecting meta-GWAS in humans. This method facilitated mapping of processing and end-use quality phenotypes from advanced breeding lines (n = 4,095) of the CIMMYT bread wheat breeding program from 2009 to 2014. Using the meta-GWAS we identified marker-trait associations, allele effects, candidate genes, and can select using markers generated in this process. Finally, the scope of this approach can be broadly applied in ‘breeding-assisted genomics’ across many crops to greatly increase our functional understanding of plant genomes.

## Introduction

The human population is growing exponentially with projections predicting greater than 9 billion people by the year 2050. Currently global bread wheat (*Triticum aestivum* L.) consumption supplies nearly 16 g of protein per capita daily and is quickly increasing in urban areas and least developed countries, which are predicted to have the largest population increases [1]. Feeding a larger, more urban population will require an increase in wheat production, which must be achieved with less land and water resources than are currently available, compelling an intersection of improved agronomic practices and crop varieties. Along with this increased production, there is also growing demand to produce end-use optimized, more nutritive, and higher-quality wheat products. The International Maize and Wheat Improvement Center (CIMMYT), breeds wheat varieties focused on markets and climates found in developing countries [2] and influences a majority of global wheat germplasm [3]. CIMMYT is breeding to achieve better wheat quality while increasing yield and enhancing disease resistance, with increased measures to incorporate genomic information to their breeding activities [4–7].

**Fig 1:**
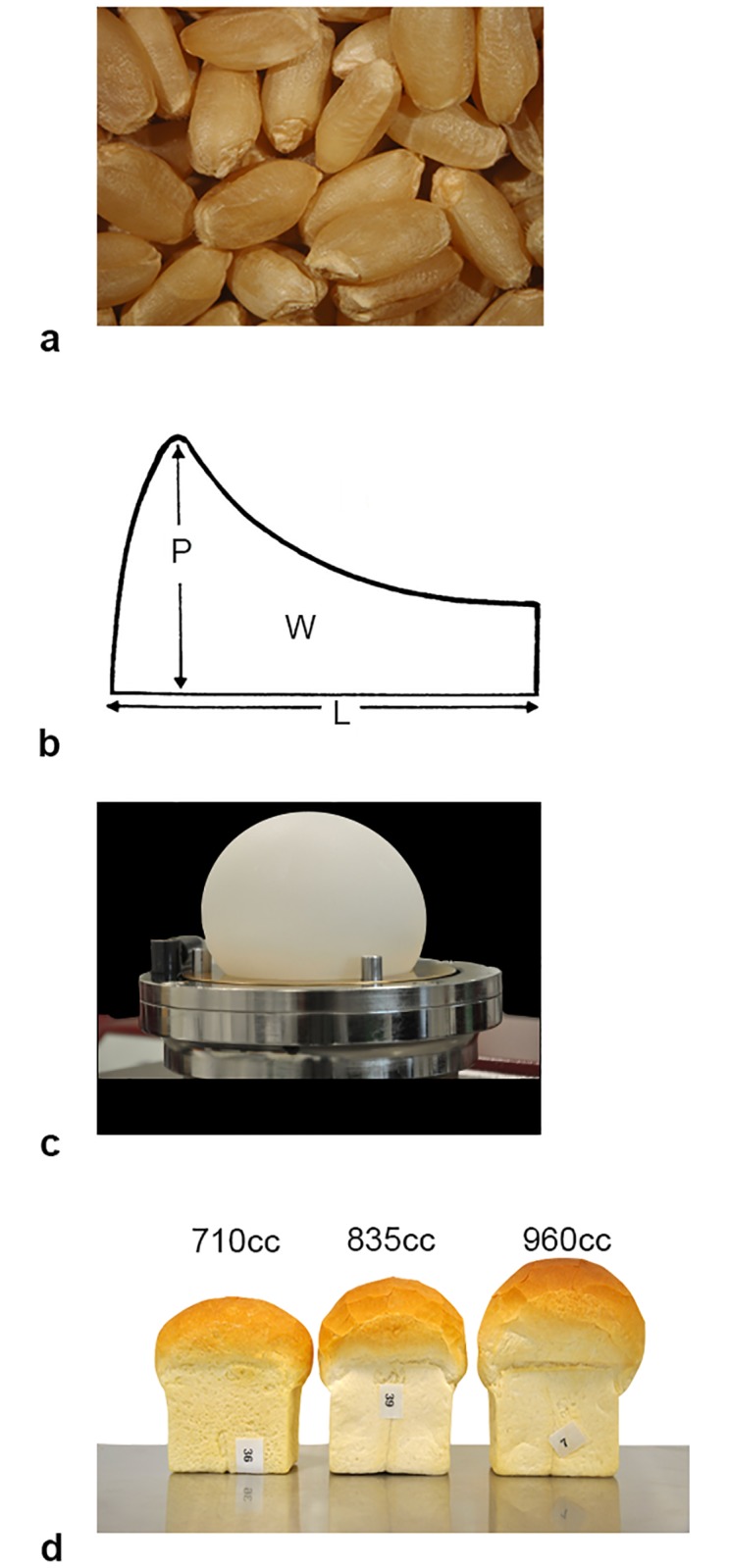
Demonstration and quantification of grain, dough and loaf volume tests. a) Grain samples for protein testing and milling, b) Alveograph example demonstrating that P/L is the height to width ratio which measures extensibility and W, area under the curve measures dough strength. These are measured on c) dough tested in forced air method of Alveograph. Loaf volume test is represented in d) with breeding lines of various volumes demonstrated.

Bread wheat flour is traditionally used for a variety of products, each with a specific profile, including protein quantity and quality [8, 9], for optimal baking in the home or industry [10]. To determine the wheat quality profile several measurements of wheat grain, flour, dough, and final products must be assessed on wheat lines within breeding programs (Fig 1) [10]. However, this testing is greatly limited by the amount of seed available and cost.

Grain tests can be done on small scale, quickly and cost effectively, making high-throughput implementation possible. However, dough rheology and end-use tests require large quantities of grain for milling into flour, restricting their implementation to advanced stages in a breeding pipeline. Finally, small loaves of bread are produced and loaf volume is measured as an indicator of product performance (Fig 1). Since these tests cannot be assessed until late in the breeding cycle marker assisted selection (MAS) could be beneficial for these traits. Flour quality has long been attributed mainly to grain storage proteins [8, 9], however, genetic identification of these proteins and others [11] related to quality traits does not fully explain the quantitative quality traits. Therefore, further understanding of genetic architecture of wheat quality is necessary.

To determine genomic architecture of quantitative traits, genetic mapping with complex structured populations [12, 13] have become common, but are limited in breeding programs due to time and resources. GWAS, alternatively, does not require structured mating, instead, large, diverse samples of individuals are used to associate genomic markers to phenotypic variation, with population structure and kinship utilized to reduce spurious associations [14]. Statistical power can be strengthened by combining results from several populations that have been studied separately through meta-analysis. Meta-GWAS studies have been utilized to detect genetic risk loci for several diseases in humans which are heavily impacted by environment or genotype-by-environment interactions [15]. Meta-studies of previously detected QTL have been utilized in wheat [16–19] and can be useful for condensing results over several studies and determining most effective loci over space and time. However, powerful meta-GWAS studies have not previously been used within a breeding program to identify QTL and immediately implement genomics-assisted breeding.

GWAS has been utilized to detect marker associations with wheat quality [20, 21], however, dough rheology traits measure by Mixograph and Alveograph, and loaf volume have not been investigated, likely due to the significant cost of these tests necessary to the breeding process. In contrast to maximizing the potential of association mapping using a diversity panel of germplasm, we present a more cost-effective meta-analyzed GWAS relying on data already generated in the breeding program.

## Materials and methods

Wheat lines used in association mapping for wheat quality were materials from preliminary and advanced yield trials of the CIMMYT bread wheat breeding program between 2009 and 2014. All wheat lines were grown in Ciudad Obregon, Sonora, Mexico, in at least one year, under full irrigation. Site-years were treated individually for the QK-Mixed model GWAS and were considered eligible for analysis if there were greater than 200 entries tested. Best materials for agronomic and quality traits were advanced in the breeding program and grown and tested a second year under full irrigation. The full set for association mapping, n = 4,095 entries, included both replicated and non-replicated entries to increase the size of the association mapping panel and show validity of the Meta-GWAS method. Materials were also utilized in genomic selection in Battenfield, et al. [7].

Wheat processing and end-use quality phenotypes for thousand kernel weight (TKW) grain protein (GRNPRO), Alveograph W (ALVW) and PL^-1^ (ALVPL), and pup loaf volume (LOFVOL), were measured according to AACC [22] methodology with minor modifications for throughput. Grain morphological characteristics were evaluated with digital image system SeedCount SC5000 (Next Instruments, Australia) and weighed to obtain TKW (g). Grain protein (GRNPRO) and moisture content were determined by near-infrared spectroscopy (NIRS), using NIR Systems 6500 (Foss, Denmark) by the official methods of the American Association of Cereal Chemists (AACC) 39–10 and 39–00, respectively [22]. GRNPRO was reported at 12.5% moisture basis. Grain samples were tempered and milled using Brabender Quadrumat Jr. (C. W. Brabender OHG, Germany). Dough rheology was assessed using the Chopin Alveograph (Tripette & Renaud, France), AACC method 54-30A [22]. These methods were adjusted to allow for optimized water content based on Solvent Retention Capacity, as in Guzmán, et al. [23]. Dough strength, work value under the curve (ALVW), and tenacity vs. extensibility, the ratio of height to length of the curve (P/L, ALVPL), were measured using Alveograph. Bread was baked to test end-use productivity as pan bread with AACC method 10–09 [22]. Pup loaf baking also utilized the Guzmán, et al. [23] adjustment for optimal water absorption. Bread loaf volume (LOFVOL) was measured by rapeseed displacement in accordance with AACC method 10–05.01 [22]. Phenotypic assessments and further characterization of these lines is found in Battenfield et al. [7].

Tissue was collected from five plants per wheat line and DNA was extracted with a modified CTAB protocol [24]. DNA was quantified, normalized, digested with two enzymes, *Pst1* and *Msp1*, ligated with barcoded adapters, amplified, and then sequenced as in the protocol of Poland, et al. [25]. DNA sequence analysis was conducted using TASSEL 5 GBS v2 pipeline [26]. GBS sequence tags were aligned to the *Triticum aestivum* IWGSC genome assembly version 2.25 [27] and indexed using Bowtie2 version 2.2.4 [28]. Bowtie2 was used to align GBS tags to the wheat genome assembly using the–*very-sensitive-local* option. SNPs were named by chromosome pseudo base pair position from the IWGSC 2.25 and numerically coded for major, minor, heterozygous, or missing classes. SNPs were then curated in JMP-Genomics 7.1 (SAS, Cary, NC) to maintain maximum data accuracy with the large amounts of missing data found using genotyping-by-sequencing. Individuals with greater than 35% missing data were removed from further analysis. Markers with greater than 25% missing data, greater than 20% percent heterozygous, or less than 5% minor allele frequency were also removed. Polymorphism information content was calculated for each marker (S1 Fig). Linkage disequilibrium (LD) was plotted and markers were removed which showed excessive LD over long genomic distances (S2 Fig). The final annotated and curated set of SNPs was aligned with PopSeq [29] to determine cM position of the markers in the ‘Synthetic W7984’ by ‘Opata M85’ doubled haploid population [30] to maintain consistency of mapping positions. Sequence and map information for GBS tags utilized in mapping is provided in S1 Table.

Population and cryptic relationship structure among individuals were investigated from the genomic data. These were added to the association mapping analysis as covariates to help prevent spurious associations [14]. Principal component analysis was conducted in JMP-Genomics 7.1 (SAS, Cary, NC) to estimate a population structure matrix, Q. Cryptic relationship (kinship) between individuals was also estimated via identity by descent method [31] in JMP-Genomics 7.1, resulting in the K matrix. Q and K principle component analyses are available in supplement (S3 Fig).

Association mapping for processing and end-use quality phenotypes was conducted using a Q-K mixed model [14] in JMP-Genomics 7.1 for each site-year with false discovery rate (FDR) multiple testing correction applied [32]. Estimated SNP effects and standard errors from each site-year marker-trait association were combined using a GWAS meta-analysis with an inverse-variance and fixed effects model where each site-year was treated as a fixed effect. Multi-year marker-trait associations were corrected for multiple testing using FDR [32] since marker effects had no prior correction. Probabilities were transformed using negative log10(*p-value*), and statistical significance was declared at *p* < 0.001. All significant marker-trait associations are reported in S2 Table.

Haplotype analyses were conducted for multiple significant markers present in narrow genetic distances with similar impact on trait of interest. Haplotype phases and probabilities for each individual within years were estimated using JMP-Genomics 7.1, and the haplotype probabilities were regressed against all traits within each year. Estimated haplotype trait means and standard errors from haplotype trend regressions for each year were combined using an inverse-variance and fixed effects meta-analysis model and *p*-values were corrected with FDR [32].

Candidate genes were identified through previous literature reports with named genes in the wheat gene catalog. If no named wheat quality QTL were identified in a region, a BLAST search was conducted within the haplotype boundaries. Predicted annotations most likely to have impact on grain fill, protein deposition, or grain storage proteins were reported.

## Results

To apply genomic tools to the CIMMYT wheat breeding program we utilized genotyping-by-sequencing (GBS) [25] and discovered a total of 1,625 high-quality SNP markers across 4,095 breeding lines from the program. Polymorphic markers were distributed throughout the genome with mean 93, 116, and 23 SNPs per chromosome in the A-, B-, and D- genomes, respectively (S1 Fig).

**Fig 2:**
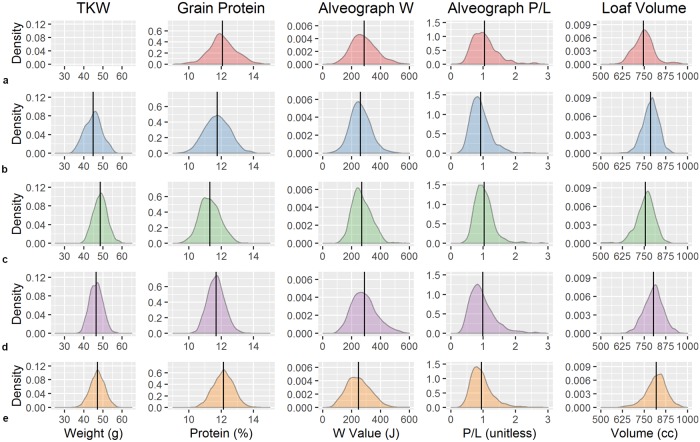
Distributions of phenotypic traits over time for thousand kernel weight (TKW, no data available in 2010), grain protein, Alveograph W and P/L, and loaf volume in a) 2010, b) 2011, c) 2012, d) 2013, and e) 2014.

**Fig 3:**
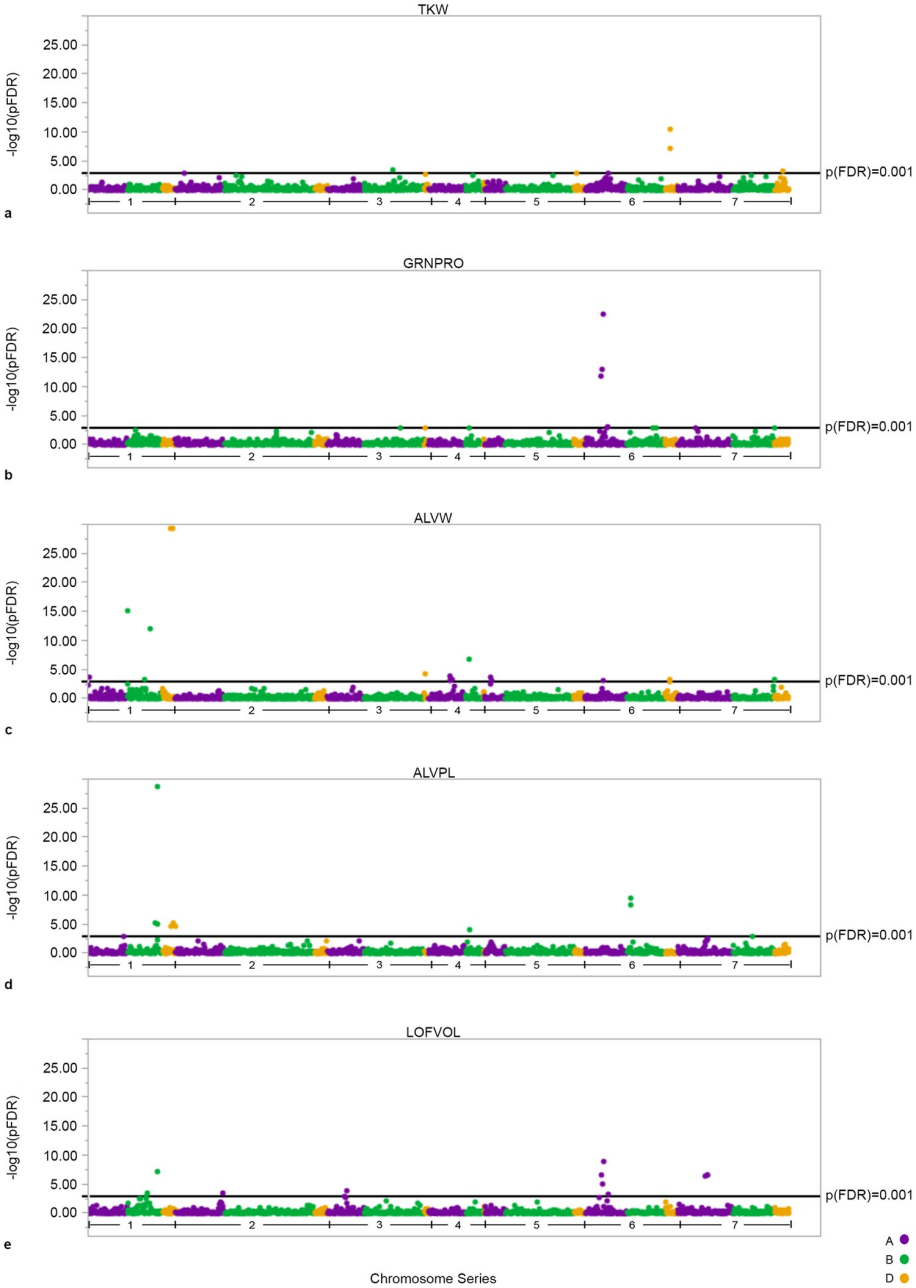
Manhattan plots of thousand kernel weight (TKW), grain protein (GRNPRO), Alveograph W (ALVW) and P/L (ALVPL), and loaf volume (LOFVOL) traits. Homeologous chromosomes are identified by number, and color separates the genome where purple is A, green is B, and yellow is D.

To capitalize on the vast amount of valuable phenotypes generated during the breeding process (Fig 2), we implemented a novel meta-GWAS approach to understand the genetic architecture of wheat quality. In this approach, QK-mixed model GWAS [14] were conducted within each year, and meta-analysis was applied over years. This new statistical approach was necessary because of the highly unbalanced nature of the data generated in breeding programs; lines were tested in only a single year with a single replication, reflecting the challenge of GWAS in humans. Fifty-two false-discovery rate corrected significant meta-marker-trait associations were found (*p* < 0.001) covering 40 unique SNPs (Fig 3; S3 Table). Seventeen significant SNPs co-localized to seven genomic regions, and an analysis of these SNPs as haplotypes was conducted, which resulted in allele identification for each line in the breeding program and quantification of allele frequencies (Table 1). Haplotypes were regressed over all phenotypes within years, then subjected to meta-analysis across years in order to determine the overall effect of the alleles within the breeding program and indicate the effects of selection across phenotypes (Fig 2).

**Table 1:** Haplotype frequencies.

POPSEQ		GBS SNP ID		Haplotype Frequencies					
Chr	cM	Haplotype borders	# SNPs	2010	2011	2012	2013	2014	Composite
1D	73.3	S3_108397610—S3_113356875	2	23.4%	1.9%	2.8%	12.2%	13.7%	8.8%
				76.2%	97.5%	96.2%	85.7%	84.9%	89.9%
4A	106–108	S10_201716835—S10_203231427	3	33.5%	46.2%	42.3%	43.6%	42.0%	42.9%
				61.1%	47.8%	51.6%	46.4%	51.1%	50.0%
6A	49–54	S16_19072856—S16_33226117	3	15.4%	22.0%	16.3%	27.1%	15.9%	19.9%
				67.7%	65.5%	71.4%	58.2%	65.6%	65.3%
6A	61–63	S16_143466155—S16_150663555	2	53.6%	44.2%	45.5%	50.6%	36.7%	44.4%
				46.4%	46.4%	48.1%	38.0%	46.3%	44.9%
6B	22.4	S17_5974923—S17_6513799	2	7.5%	21.5%	14.9%	18.8%	10.0%	15.5%
				91.2%	76.8%	83.7%	79.8%	88.5%	83.0%
6D	78–82	S18_20387611—S18_111388404	2	30.0%	34.5%	33.4%	39.9%	31.8%	34.4%
				56.8%	56.4%	54.6%	42.4%	42.3%	49.2%
7A	93.3	S19_78415889—S19_112027332	3	28.4%	41.5%	39.0%	35.2%	23.6%	34.0%
				60.3%	48.8%	50.4%	52.5%	66.1%	55.3%

**Fig 4:**
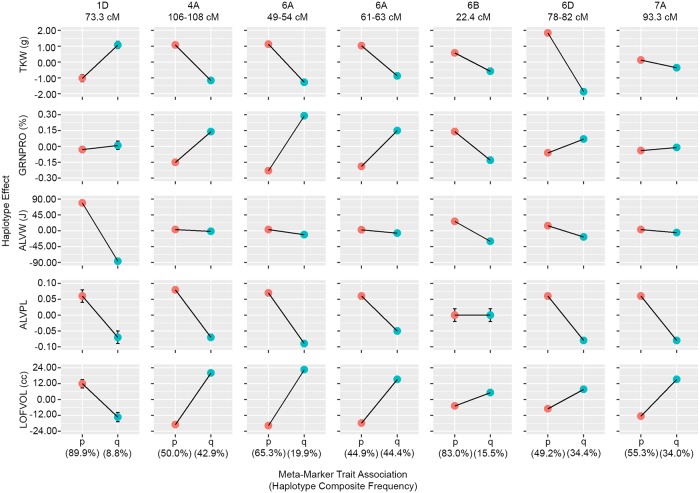
Meta-marker trait associations for seven significant multi-trait associations. Meta effect of each haplotype is displayed with marker frequencies. Effects are demonstrated for thousand kernel weight (TKW), grain protein (GRNPRO), Alveograph W (ALVW) and P/L (ALVPL), and loaf volume (LOFVOL) traits.

GRNPRO is highly correlated with dough strength [33], LOFVOL [34], and overall baking quality [35]. In industrial food manufacturing, protein is often added as vital wheat gluten to increase functionality of dough, but represents an added cost to that industry. Here we identified several significant regions controlling grain protein concentration in meta-GWAS analysis (Fig 2, S3 Table), and four significant meta-haplotypes that demonstrate similar trends across the phenotypes. These meta-haplotype results indicate that GRNPRO is positively correlated with LOFVOL (Fig 4). However, GRNPRO is often negatively correlated with yield, which is corroborated by the observed negative correlation to TKW for several alleles.

In an effort to further increase genomic information in the breeding program, candidate genes were identified for these significant meta-haplotypes (Table 1). Several genes involved in photosynthesis and starch synthesis were located on chromosome 4A between 105–107 cM. Two QTL with major effect on GRNPRO were found on chromosome 6A (Fig 4). The first haplotype located at 49–54 cM on chromosome 6A maps near *NAM-A1*, which impacts senescence timing, TKW, and GRNPRO [36], and is homeologous to *Gpc-B1* [37]. The second haplotype located at 61–63 cM is located near *TaGW2*, which impacts TKW and kernel width [38]. Additionally, we identified a meta-haplotype at 81.58 cM on chromosome 6D, which localizes near homeologous genetic sequence to *TaGW2*, which could possibly represent *TaGW2-D1* with further study. Since these QTL all impact TKW, a component of yield, as well as GRNPRO and LOFVOL (Fig 4), breeding decisions should be carefully weighed regarding the tradeoffs presented by these loci.

Dough strength and extensibility are important characteristics to determine whether flour is best suited to be used in home or industrial processes and which is the optimal end-use product. For example, a strong, extensible dough would be optimal for industrial bread making, whereas a medium-strong, extensible dough may be better for home production of flat breads [10]. Alveograph testing measures dough strength, area under curve (ALVW), and extensibility, height to width ratio (ALVPL); by blowing air into dough (Fig 1). Large effects on dough rheology have long been associated with the presence of specific grain storage protein subunits [9], which alter protein quality without significant changes to protein quantity. Here we report significant associations for dough rheology, and LOFVOL relating to storage proteins. Specifically, high molecular weight glutenin, *Glu-D1*, is the candidate gene for the QTL on chromosome 1D, which has the largest effect on dough rheology in this study (Fig 4). Additionally, a QTL on 6B was found to have large effect on ALVW and several predicted nitrate transporter annotations were found in this region, which may impact grain proteins.

Final LOFVOL is a complex, but heritable trait that is impacted by quality and quantity of the storage proteins present in the flour, as well as by non-protein factors [7]. Here we display the combined results of meta-GWAS from measurement of 4,095 empirically baked loaves of bread. Our results indicate LOFVOL is impacted by all aforementioned QTL (Fig 4). Additionally, a QTL for ALVPL and LOFVOL was found on chromosome 7A (93 cM), located near the wheat bread making (*wbm*) gene [39]. The allele favoring higher LOFVOL at this locus is a minor allele within the CIMMYT breeding program, but CIMMYT sources of this favorable allele are described in Guzmán, et al. [40]and MAS can be utilized.

## Discussion

Meta-GWAS is a powerful, novel method, which allowed the largest genetic study of wheat quality to date (n = 4,095). Using meta-GWAS GBS markers associated with major processing and end-use quality traits were discovered for all traits examined. However, the more applicable outcome was combining single marker trait associations into haplotype makers. Haplotype QTL facilitated the ability to reliably test for presence or absence, designate a window where candidate genes should be explored, and allowed for tests of QTL effect on all other traits.

Here we demonstrate the implications of this methodology on seven meta-haplotypes which impact wheat processing and end-use quality as well as grain thousand kernel weight. In the CIMMYT bread wheat breeding program, all QTL, except one, had been selected where the more frequent allele favored increased TKW, but decreased final loaf volume. This is unsurprising as the primary goal of the breeding program is to increase grain yield while continuing to make acceptable food products. The one exception of QTL predominantly selected for increased ALVW with decreased TKW, *GluD1*, is well known for majorly impacting dough strength, and is commonly selected using marker assisted selection in breeding programs [8, 9].

As many QTL documented in this study impact TKW, there is potential for use as selection targets for breeding or gene editing for increased yield potential. The majority of these QTL seem to impact starch synthesis or grain filling [36–38]. So, while these increases may be beneficial in raising the quantity of wheat produced, it seems that in order for functional products to continue to be made, care will need to be taken to ensure quality and quantity of functional protein remain in the flour. Adversely, the 7A *wbm* QTL remains protein and grain size neutral, while selection impact gluten extensibility and loaf volume [39]. As the increased loaf volume allele is currently less frequent in the CIMMYT bread wheat breeding program, there is ability to select this QTL to increase loaf volume without selecting for decreased grain size or protein [40].

In this study we show that meta-GWAS can be used as a powerful approach for insight to the genetic basis of important traits in a breeding program. This will enable more robust and genetically informed breeding approaches to compliment QTLs with designed crossing strategies and marker-assisted selection. Given the unbalanced testing and highly dynamic nature of breeding programs, this approach to ‘breeding-assisted genomics’ can be applied to other traits and species, allowing for immediate breeding for beneficial alleles in parallel to uncovering the genetic basis of important traits. This advantage of decreased time from marker-trait association to implementation in breeding could assist in the rapid development of crop varieties adapted to changing climates while simultaneously possessing quality characteristics for existing and emerging food markets.

## Supporting information

S1 Fig Genomic distribution of polymorphic information content (PIC) of markers used in this study. For each chromosome the physical position of the marker in base pairs shown with the respective PIC value on color scale.
(TIF)

S2 Fig Linkage disequilibrium (LD) over physical distance for all chromosomes. Pairwise LD among all markers used in this study grouped by chromosome with physical distance in bp between pairs of markers on x-axis and correlation coefficient (R^2^) on y-axis.
(TIF)

S3 Fig Principle components of the population and relationship structures. Three-dimensional display of principle components for a) population structure and b) cryptic relationship structure, also presented with two-dimensional outline of principle components for c) population structure and d) cryptic relationship structure. Scree plots demonstrate variation explained by each principle component in e) population structure and f) cryptic relationship structure.
(TIF)

S1 Table Position of GBS markers used in this study. GBS tags were aligned to Chapman, et al. population to identify chromosome and cM positions. MarkerName: given name of the respective marker based on the IWGSC draft sequence 2.25 position; Strand: forward (+) or reverse (-) strand; Tag: DNA sequence of genotyping-by-sequencing tag where SNP was identified; POPSEQ Chrom: chromosome designation based on PopSeq position assignment; POPSEQ cM: genetic position in centiMorgans based on PopSeq position assignment.
(CSV)

S2 Table Significant meta-GWAS SNPs. Marker ID is the GBS SNP name in this study. Chromosome and position are listed as identified by Bowtie 2 aligned to IWGSC 2.25. POPSEQ chromosome and position are from markers which aligned to the Chapman, et al. population. Trait is the phenotypic trait of interest. Significant effect and standard error of the effect are listed along with the negative log10 probability of the significant marker-trait association corrected with False Discovery Rate.
(PDF)

S3 Table Haplotype effects and standard errors. TKW: thousand kernel weight; GRNPRO: grain protein; ALVW: Alveograph W; ALVPL: Alveograph P/L; LOFVOL; loaf volume. Significance levels: *** *p* < 0.001; ** *p* < 0.01; * *p* < 0.05.
(PDF)
