# Supplementary figures and images for: Breeding-assisted genomics: Applying meta-GWAS for milling and baking quality in CIMMYT wheat breeding program
Source: PLoS One. 2018 Nov 29;13(11):e0204757. doi: 10.1371/journal.pone.0204757 (PMC6264898; doi:10.1371/journal.pone.0204757)

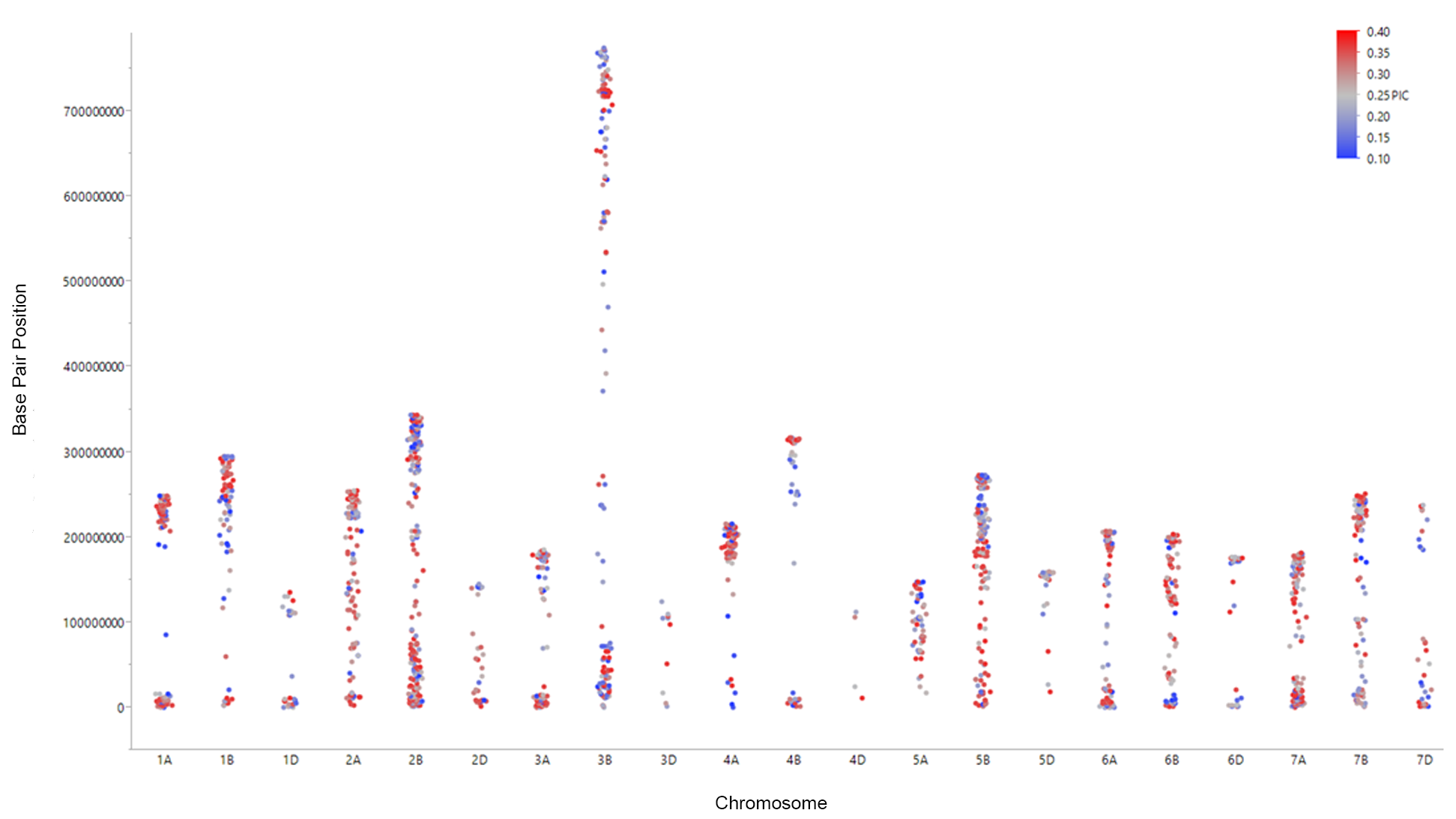

Supplement: S1 Fig — For each chromosome the physical position of the marker in base pairs shown with the respective PIC value on color scale. (TIF) [file pone.0204757.s001.tif]

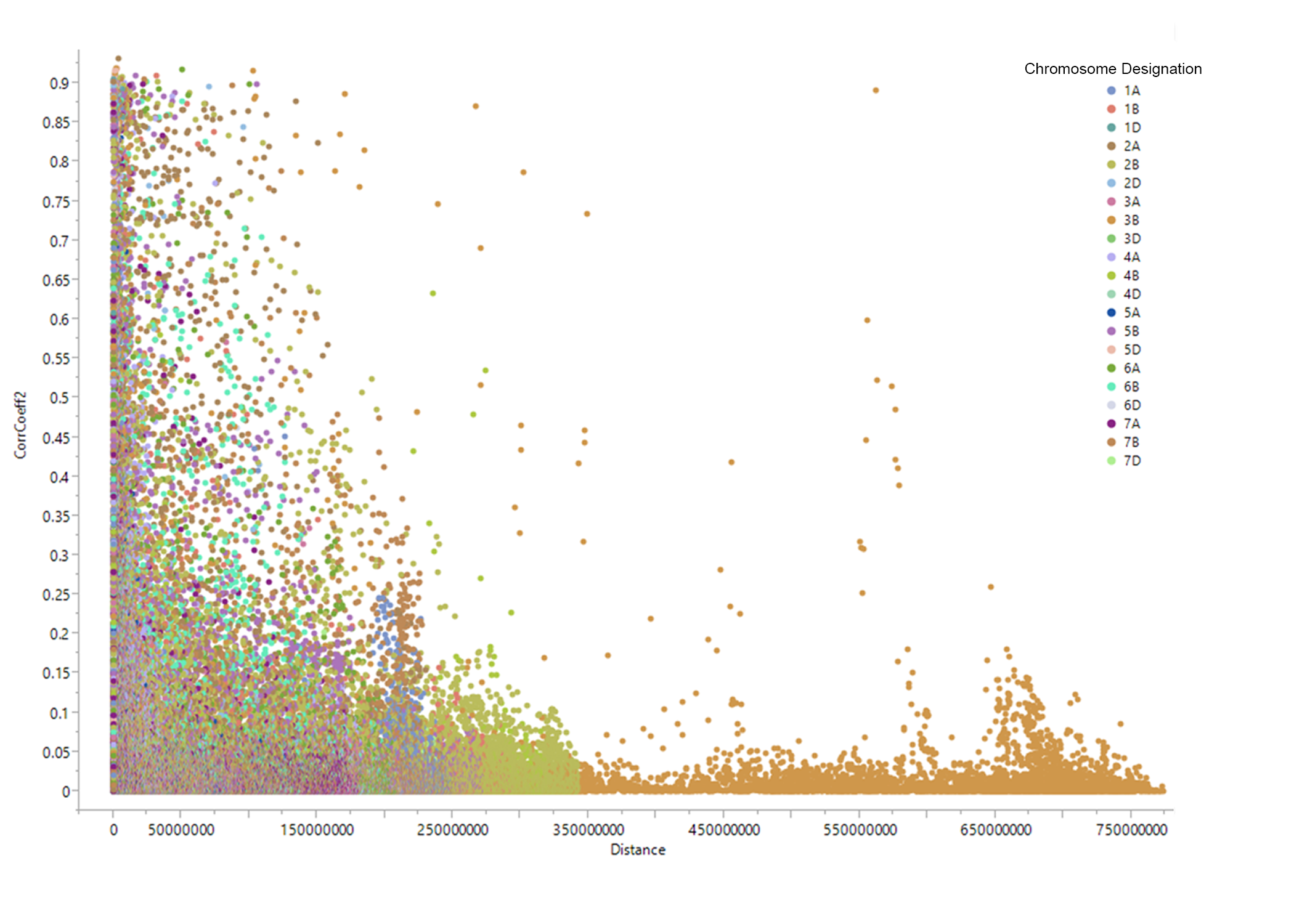

Supplement: S2 Fig — Pairwise LD among all markers used in this study grouped by chromosome with physical distance in bp between pairs of markers on x-axis and correlation coefficient (R2) on y-axis. (TIF) [file pone.0204757.s002.tif]

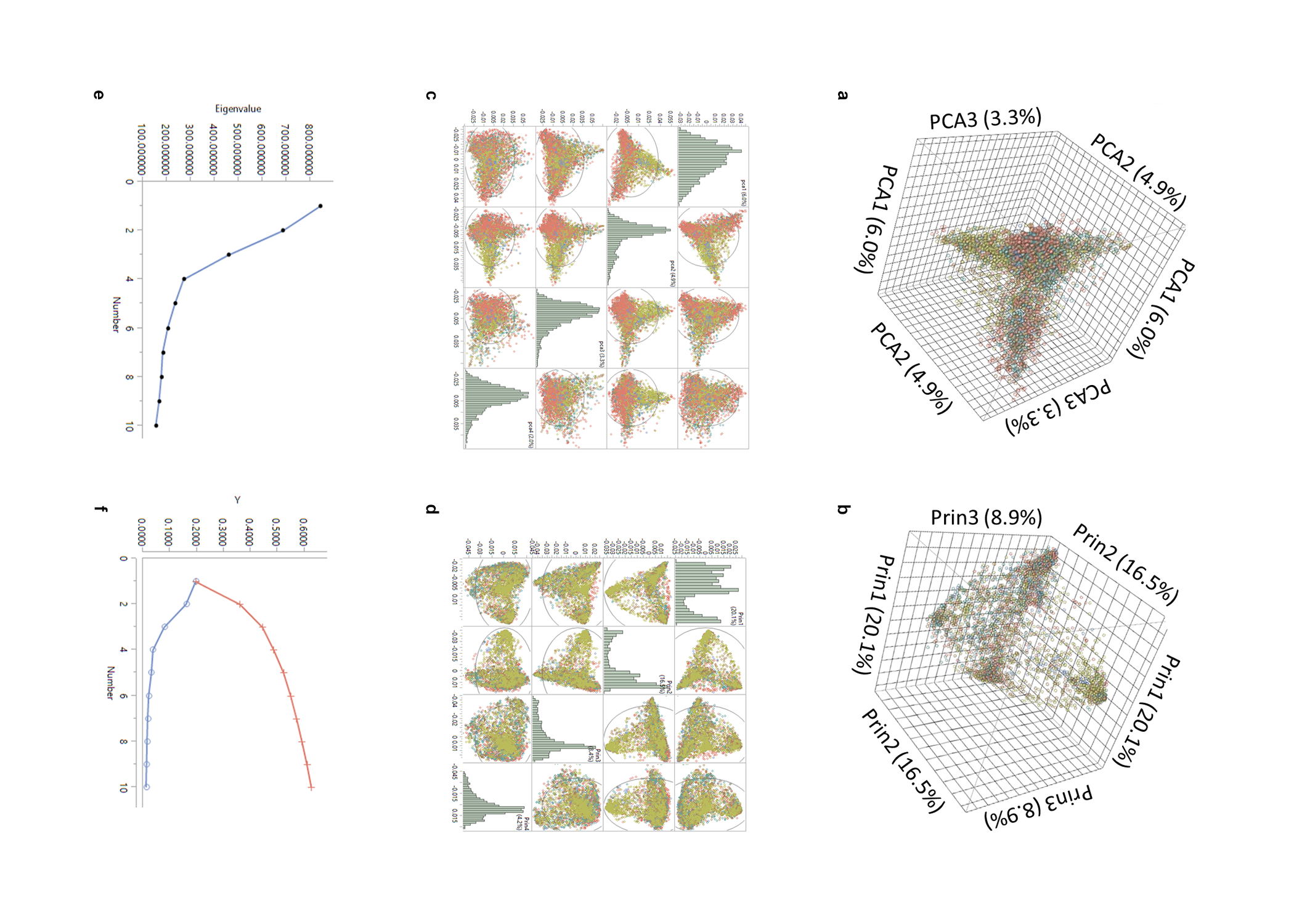

Supplement: S3 Fig — Three-dimensional display of principle components for a) population structure and b) cryptic relationship structure, also presented with two-dimensional outline of principle components for c) population structure and d) cryptic relationship structure. Scree plots demonstrate variation explained by each principle component in e) population structure and f) cryptic relationship structure. (TIF) [file pone.0204757.s003.tif]
