# Supplementary material for: Breeding-assisted genomics: Applying meta-GWAS for milling and baking quality in CIMMYT wheat breeding program
Source: PLoS One. 2018 Nov 29;13(11):e0204757. doi: 10.1371/journal.pone.0204757 (PMC6264898; doi:10.1371/journal.pone.0204757)
Supplement: S2 Table — Marker ID is the GBS SNP name in this study. Chromosome and position are listed as identified by Bowtie 2 aligned to IWGSC 2.25. POPSEQ chromosome and position are from markers which aligned to the Chapman, et al. population. Trait is the phenotypic trait of interest. Significant effect and standard error of the effect are listed along with the negative log10 probability of the significant marker-trait association corrected with False Discovery Rate. (PDF) [file pone.0204757.s005.pdf]

Supp. Table 2: Significant Meta-GWAS SNPs

Marker ID	IWGSC 2.25 Chr.	IWGSC 2.25 bp	POPSEQ Chr.	POPSEQ cM	Trait	Overall Effect	Overall SE	Overall -Log10 (pFDR)
Loci_S1_1246860	1A	1246860	1A	1.79	ALVW	-8.06	1.66	3.71
Loci_S1_245341120	1A	245341120	1A	165.46	ALVPL	-0.03	0.01	3.08
Loci_S2_2532201	1B	2532201			ALVW	-15.20	1.73	15.32
Loci_S2_247110912	1B	247110912			ALVW	-11.44	2.47	3.33
Loci_S2_258464672	1B	258464672	1B	93.52	LOFVOL	-5.84	1.23	3.51
Loci_S2_266250969	1B	266250969	1B	97.83	ALVW	9.99	1.26	12.08
Loci_S2_276759258	1B	276759258	1B	111.09	ALVPL	-0.05	0.01	5.36
Loci_S2_282749164	1B	282749164	1B	125.88	ALVPL	0.11	0.01	28.93
Loci_S2_282749164	1B	282749164	1B	125.88	LOFVOL	-6.90	1.06	7.36
Loci_S2_282801547	1B	282801547	1B	125.88	ALVPL	0.05	0.01	5.16
Loci_S3_108397610	1D	108397610	1D	73.3	ALVW	48.27	2.61	29.46
Loci_S3_108397610	1D	108397610	1D	73.3	ALVPL	0.07	0.01	4.68
Loci_S3_113356875	1D	113356875	1D	73.3	ALVW	46.16	2.57	29.46
Loci_S3_113356875	1D	113356875	1D	73.3	ALVPL	0.07	0.01	5.33
Loci_S3_134641057	1D	134641057	1D	154.66	ALVPL	-0.04	0.01	4.71
Loci_S4_253161415	2A	253161415	2A	120.3	LOFVOL	-7.04	1.49	3.50
Loci_S7_70434692	3A	70434692	3A	60.62	LOFVOL	-7.36	1.49	3.87
Loci_S8_511291591	3B	511291591			TKW	0.72	0.14	3.67

Loci_S8_652811064	3B	652811064	3B	79.46	GRNPRO	0.06	0.01	3.01
Loci_S9_50810552	3D	50810552	3D	71.95	ALVW	-7.55	1.44	4.42
Loci_S9_50810552	3D	50810552	3D	71.95	GRNPRO	-0.06	0.01	3.02
Loci_S10_201716835	4A	201716835	4A	105.75	ALVW	11.46	2.30	3.92
Loci_S10_202066152	4A	202066152	4A	107.52	ALVW	11.38	2.50	3.27
Loci_S10_203231427	4A	203231427	4A	107.52	ALVW	-11.26	2.45	3.33
Loci_S11_9212182	4B	9212182	4B	44.35	ALVPL	-0.04	0.01	4.23
Loci_S11_9212182	4B	9212182	4B	44.35	GRNPRO	-0.06	0.01	3.01
Loci_S11_9791147	4B	9791147	4B	44.35	ALVW	-14.31	2.30	6.88
Loci_S13_77029577	5A	77029577	5A	8.82	ALVW	7.99	1.63	3.76
Loci_S13_82914716	5A	82914716			ALVW	-7.06	1.57	3.20
Loci_S16_19072856	6A	19072856	6A	49.22	GRNPRO	-0.12	0.01	11.90
Loci_S16_19072856	6A	19072856	6A	49.22	LOFVOL	-7.86	1.28	6.62
Loci_S16_24998762	6A	24998762	6A	49.22	GRNPRO	-0.13	0.02	13.16
Loci_S16_24998762	6A	24998762	6A	49.22	LOFVOL	-7.70	1.40	5.15
Loci_S16_33226117	6A	33226117	6A	53.81	GRNPRO	-0.16	0.02	22.63
Loci_S16_33226117	6A	33226117	6A	53.81	LOFVOL	-9.80	1.37	8.96
Loci_S16_33226117	6A	33226117	6A	53.81	ALVW	-8.72	1.97	3.11
Loci_S16_143466155	6A	143466155	6A	60.96	GRNPRO	0.07	0.02	3.01
Loci_S16_150663555	6A	150663555	6A	63.1	LOFVOL	10.73	2.31	3.36
Loci_S16_150663555	6A	150663555	6A	63.1	GRNPRO	0.12	0.03	3.21
Loci_S17_5974923	6B	5974923	6B	22.38	ALVPL	-0.08	0.01	9.55

Loci_S17_6513799	6B	6513799	6B	22.38	ALVPL	-0.07	0.01	8.45
Loci_S17_164481606	6B	164481606	6B	78.42	GRNPRO	0.08	0.02	3.01
Loci_S18_20387611	6D	20387611	6D	77.64	TKW	0.59	0.08	10.63
Loci_S18_111388404	6D	111388404	6D	81.58	TKW	0.51	0.08	7.20
Loci_S18_111388404	6D	111388404	6D	81.58	ALVW	6.79	1.47	3.33
Loci_S19_78415889	7A	78415889			LOFVOL	-7.59	1.25	6.55
Loci_S19_101352522	7A	101352522	7A	93.26	LOFVOL	-7.64	1.25	6.62
Loci_S19_112027332	7A	112027332	7A	93.26	LOFVOL	-8.15	1.30	6.74
Loci_S20_173057509	7B	173057509	7B	79.65	ALVPL	-0.04	0.01	3.08
Loci_S20_251235369	7B	251235369	7B	172.71	ALVW	7.36	1.60	3.33
Loci_S20_251235369	7B	251235369	7B	172.71	GRNPRO	-0.06	0.01	3.01
Loci_S21_55673430	7D	55673430	7D	111.08	TKW	-0.48	0.10	3.38

Marker ID is the GBS SNP name in this study. Chromosome and position are listed as identified by Bowtie 2[36] aligned to IWGSC 2.25. POPSEQ chromosome and position are from markers which aligned to the Chapman, et al. [37] population. Trait is the phenotypic trait of interest. Significant effect and standard error of the effect are listed along with the negative log10 probability of the significant marker-trait association corrected with False Discovery Rate.
