# Supplementary material for: Breeding-assisted genomics: Applying meta-GWAS for milling and baking quality in CIMMYT wheat breeding program
Source: PLoS One. 2018 Nov 29;13(11):e0204757. doi: 10.1371/journal.pone.0204757 (PMC6264898; doi:10.1371/journal.pone.0204757)
Supplement: S3 Table — TKW: thousand kernel weight; GRNPRO: grain protein; ALVW: Alveograph W; ALVPL: Alveograph P/L; LOFVOL; loaf volume. Significance levels: *** p < 0.001; ** p < 0.01; * p < 0.05. (PDF) [file pone.0204757.s006.pdf]

Supp Table 3: Haplotype effects and standard errors

Chromosome & position	Composite Frequency	TKW (g)		GRNPRO (% content)		ALVW (J)		ALVPL		LOFVOL (cm^3)	
		Effect	SE	Effect	SE	Effect	SE	Effect	SE	Effect	SE
1D - 73.3 cM	8.8%	1.08***	0.23	0.01	0.04	-86.44***	4.82	-0.07***	0.02	-13.43***	3.44
	89.9%	-1.02***	0.22	-0.03	0.03	79.25***	4.42	0.06**	0.02	11.90***	3.17
4A - 106-108 cM	42.9%	-1.16***	0.14	0.14***	0.02	-1.65	2.54	-0.07***	0.01	20.04***	1.82
	50.0%	1.08***	0.14	-0.15***	0.02	3.55	2.55	0.08***	0.01	-19.07***	1.84
6A - 49-54 cM	19.9%	-1.27***	0.18	0.29***	0.03	-11.04***	3.22	-0.09***	0.01	22.54***	2.25
	65.3%	1.12***	0.14	-0.23***	0.02	3.29	2.65	0.07***	0.01	-19.83***	1.88
6A - 61-63 cM	44.4%	-0.88***	0.15	0.15***	0.02	-6.76	2.71	-0.05***	0.01	15.21***	1.96
	44.9%	1.03***	0.15	-0.19***	0.02	2.74	2.64	0.06***	0.01	-17.84***	1.91
6B - 22.4 cM	15.5%	-0.58**	0.20	-0.13***	0.03	-29.57***	3.38	0.00	0.02	5.27*	2.44
	83.0%	0.58**	0.19	0.14***	0.03	26.93***	3.25	0.00	0.02	-4.91*	2.34
6D – 78-82 cM	34.4%	-1.88***	0.14	0.07***	0.02	-17.31***	2.66	-0.08***	0.01	7.69***	1.94
	49.2%	1.83***	0.14	-0.06**	0.02	14.52***	2.52	0.06***	0.01	-7.00***	1.86
7A - 93.3 cM	34.0%	-0.35*	0.15	-0.01	0.02	-5.03	2.62	-0.08***	0.01	15.33***	1.91
	55.3%	0.13	0.14	-0.04	0.02	3.77	2.52	0.06***	0.01	-12.59***	1.83

Significance levels: *** $p < 0.001$; ** $p < 0.01$; * $p < 0.05$
